# Supplementary material for: Metabolic disturbances, hemoglobin A1c, and social cognition impairment in Schizophrenia spectrum disorders
Source: Transl Psychiatry. 2022 Jun 6;12:233. doi: 10.1038/s41398-022-02002-z (PMC9170776; doi:10.1038/s41398-022-02002-z)
Supplement: Supplementary file 1 — Supplemental Materials [file 41398_2022_2002_MOESM1_ESM.pdf]

# Metabolic Disturbances, Hemoglobin A1c, and Impairment in Social Cognition in Schizophrenia Spectrum Disorders

## <u>SUPPLEMENTAL MATERIALS</u>

## Supplemental Table 1: Overall Social Cognition, Metabolic Risk, and Hemoglobin A1c

| Outcome | Predictors | Std. $\beta$ | p | Adjusted R-Squared |
|---|---|---|---|---|
| Overall Social Cognition | HbA1c | -0.37 | ***<0.001*** | 0.14 |
| | | | | |
| Overall Social Cognition | HbA1c | -0.19 | ***0.006*** | 0.23 |
| | Age | -0.35 | ***<0.001*** | |
| | Sex (Male) | -0.06 | 0.31 | |
| | Asian Race | -0.08 | 0.34 | |
| | Black Race | -0.20 | 0.07 | |
| | Mixed Race | 0.08 | 0.26 | |
| | White Race | 0.03 | 0.81 | |
| | Hispanic Ethnicity | -0.10 | 0.16 | |
| | | | | |
| Overall Social Cognition | HbA1c | -0.27 | ***<0.001*** | 0.22 |
| | Duration of Illness | -0.31 | ***<0.001*** | |
| | | | | |
| Overall Social Cognition | HbA1c | -0.38 | ***<0.001*** | 0.15 |
| | BPRS Total Score | -0.15 | ***0.02*** | |
| | | | | |
| Overall Social Cognition | HbA1c | -0.18 | **0.001** | 0.53 |
| | Age | -0.30 | **<0.001** | |
| | Nonsocial Neurocog | 0.57 | **<0.001** | |
| | | | | |
| Overall Social Cognition | HbA1c | -0.27 | ***<0.001*** | 0.21 |
| | Age | -0.31 | ***<0.001*** | |
| | BMI | 0.09 | 0.18 | |
| | | | | |
| Overall Social Cognition | HbA1c | -0.16 | ***0.005*** | 0.53 |
| | Nonsocial Neurocog | 0.56 | ***<0.001*** | |
| | Age | -0.32 | ***<0.001*** | |
| | Sex (Male) | -0.06 | 0.27 | |
| | Asian Race | -0.10 | 0.16 | |

| | | | | |
|---|---|---|---|---|
| | Black Race | -0.17 | ***0.04*** | |
| | Mixed Race | 0.05 | 0.41 | |
| | White Race | -0.10 | 0.22 | |
| | Hispanic Ethnicity | -0.05 | 0.33 | |
| Overall Social Cognition | #MetabolicRisk | -0.38 | ***<0.001*** | 0.14 |
| Overall Social Cognition | #MetabolicRisk | -0.26 | ***0.002*** | 0.20 |
| | Age | -0.29 | ***<0.001*** | |
| | Sex (Male) | -0.13 | 0.08 | |
| | Asian Race | -0.13 | 0.21 | |
| | Black Race | -0.20 | 0.08 | |
| | Mixed Race | 0.08 | 0.35 | |
| | White Race | -0.01 | 0.91 | |
| | Hispanic Ethnicity | -0.08 | 0.29 | |
| Overall Social Cognition | #MetabolicRisk | -0.11 | *0.08* | 0.53 |
| | Age | -0.28 | **<0.001** | |
| | Nonsocial Neurocog | 0.60 | **<0.001** | |
| Overall Social Cognition | #MetabolicRisk | -0.38 | ***<0.001*** | 0.16 |
| | BPRS Total Score | -0.17 | **0.02** | |
| Overall Social Cognition | #MetabolicRisk | -0.13 | 0.06 | 0.54 |
| | Nonsocial Neurocog | 0.60 | ***<0.001*** | |
| | Age | -0.30 | ***<0.001*** | |
| | Sex (Male) | -0.09 | 0.12 | |
| | Asian Race | -0.16 | ***0.03*** | |
| | Black Race | -0.17 | ***0.05*** | |
| | Mixed Race | 0.03 | 0.69 | |
| | White Race | -0.12 | 0.19 | |
| | Hispanic Ethnicity | -0.07 | 0.25 | |

## Supplemental Table 2: Social Cognition Domains and Hemoglobin A1c

| Outcome | Predictors | Std. $\beta$ | p | Adjusted R-Squared |
|---|---|---|---|---|
| Emotion Processing Domain | HbA1c | -0.36 | ***<0.001*** | 0.13 |
| | | | | |
| Emotion Processing Domain | HbA1c | -0.16 | ***0.01*** | 0.27 |
| | Age | -0.43 | ***<0.001*** | |
| | Sex (Male) | -0.08 | 0.18 | |
| | Asian Race | 0.02 | 0.84 | |
| | Black Race | -0.05 | 0.65 | |
| | Mixed Race | 0.03 | 0.69 | |
| | White Race | 0.14 | 0.2 | |
| | Hispanic Ethnicity | -0.03 | 0.65 | |
| | | | | |
| Emotion Processing Domain | HbA1c | -0.14 | ***0.01*** | 0.47 |
| | Age | -0.39 | ***<0.001*** | |
| | Nonsocial Neurocog | 0.47 | **<0.001** | |
| | | | | |
| Theory of Mind Domain | HbA1c | -0.31 | ***<0.001*** | 0.09 |
| | | | | |
| Theory of Mind Domain | HbA1c | -0.22 | ***0.002*** | 0.11 |
| | Age | -0.16 | ***0.02*** | |
| | Sex (Male) | -0.06 | 0.37 | |
| | Asian Race | -0.14 | 0.14 | |
| | Black Race | -0.23 | ***0.04*** | |
| | Mixed Race | 0.12 | 0.11 | |
| | White Race | -0.09 | 0.42 | |
| | Hispanic Ethnicity | -0.15 | ***0.03*** | |
| | | | | |
| Theory of Mind Domain | HbA1c | -0.18 | ***0.001*** | 0.40 |
| | Age | -0.14 | ***0.01*** | |
| | Nonsocial Neurocog | 0.55 | **<0.001** | |
| | | | | |
| Social Perception Domain | HbA1c | -0.27 | ***<0.001*** | 0.07 |
| | | | | |
| Social Perception Domain | HbA1c | -0.12 | 0.07 | 0.16 |
| | Age | -0.22 | ***0.001*** | |
| | Sex (Male) | -0.02 | 0.75 | |
| | Asian Race | -0.23 | ***0.01*** | |
| | Black Race | -0.40 | ***<0.001*** | |
| | Mixed Race | 0.18 | ***0.01*** | |
| | White Race | -0.15 | 0.2 | |
| | Hispanic Ethnicity | -0.22 | ***0.001*** | |
| | | | | |
| Social Perception Domain | HbA1c | -0.14 | ***0.02*** | 0.33 |
| | Age | -0.17 | ***0.004*** | |
| | Nonsocial Neurocog | 0.50 | **<0.001** | |

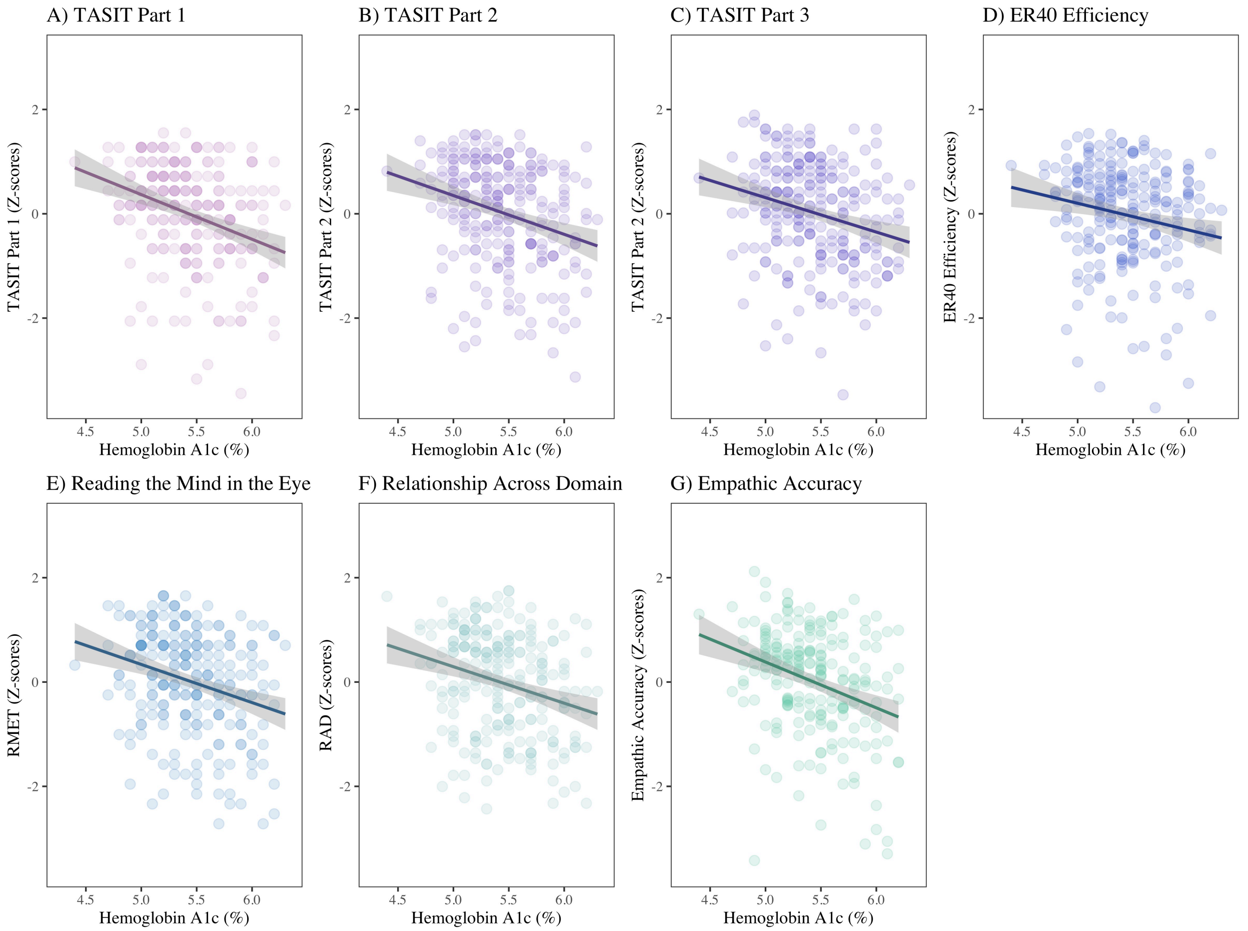


**Supplemental Figure 1 – Individual Social Cognition Tasks and Hemoglobin A1c:** Relationships between standardized z-scores for each social cognition task and HbA1c level are shown. (A) The Awareness of Social Inference Task (TASIT) Part 1, β=-0.31, p<0.001, $R^2$ 0.09. (B) TASIT Part 2, β=-0.28, p<0.001, $R^2$ 0.08. (C) TASIT Part 3, β=-0.26, p<0.001, $R^2$ 0.07. (D) Penn Emotion Recognition 40 (ER40) Efficiency, β=-0.19, p=0.003, $R^2$ 0.03. (E) Reading the Mind in the Eyes Test (RMET), β=-0.27, p<0.001, $R^2$ 0.07. (F) Relationship Across Domains Task (RAD), β=-0.27, p<0.001, $R^2$ 0.07. (G) Empathic Accuracy (EA), β=-0.31, p<0.001, $R^2$ 0.09. Standardized β coefficients are reported.

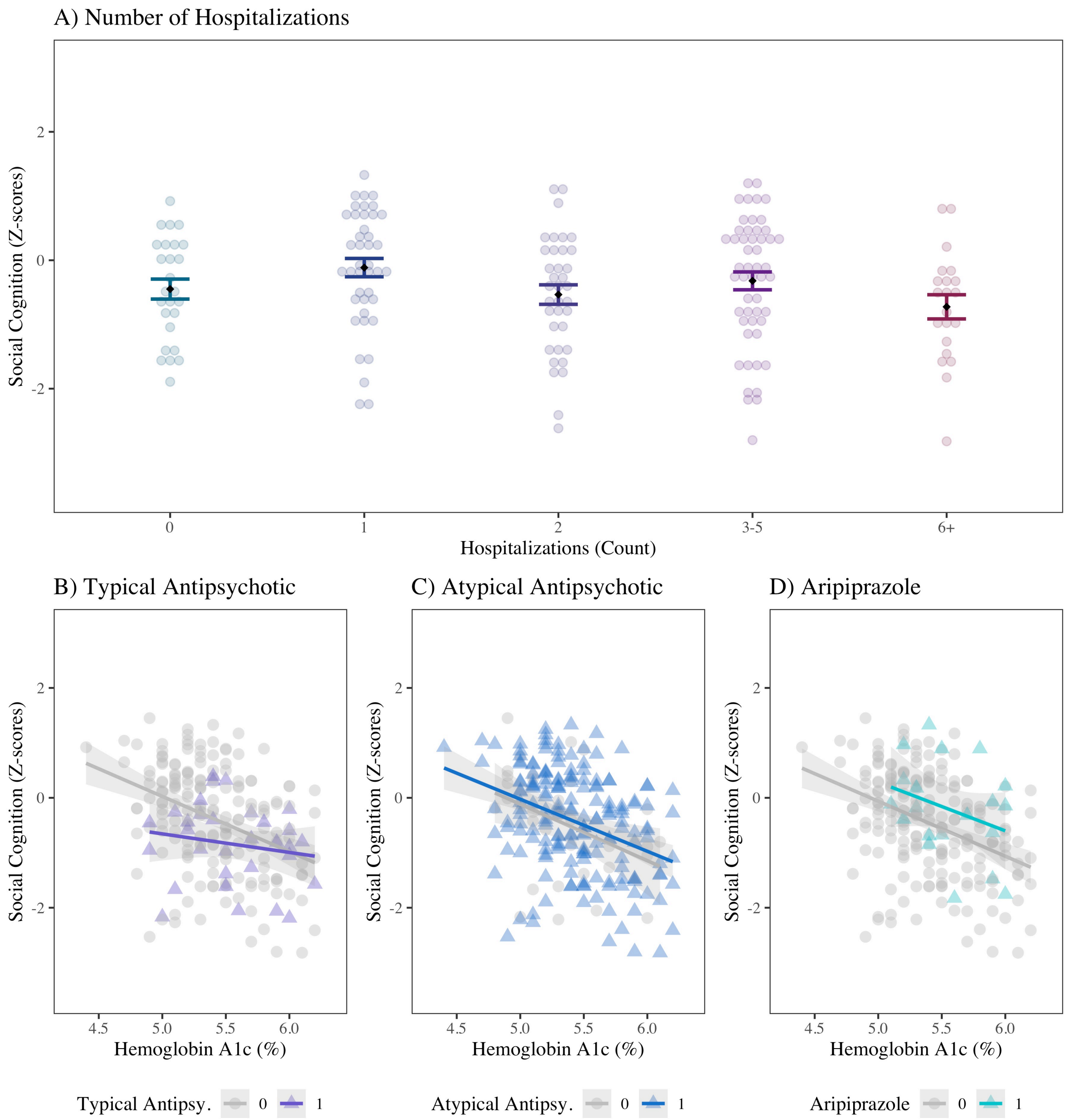


**Supplemental Figure 2 – Hospitalization and Antipsychotic Treatment Effects**: (A) Number of prior hospitalizations was not related to aggregate social cognition scores (β=0.10, p=0.16). (B) Treatment with typical antipsychotic medications had a trend level effect on the relationship between HbA1c and social cognition (β=0.12, p=0.055) , but the negative relationship between HbA1c and social cognition remained highly significant. (C) Treatment with an atypical antipsychotic (β=0.11, p=0.47) did not significantly affect the relationship between HbA1c and overall social cognition score. (D) Treatment with aripiprazole was associated with significant increase in overall social cognition score (β=0.13, p=0.047) but did not disrupt the correlation between HbA1c and social cognition. Not shown, we also examined clozapine and olanzapine together as one group, given the similarity of their metabolic effects. There was no significant effect of treatment with either clozapine or olanzapine on the relationship between HbA1c and social cognition (β=-0.09, p=0.17). Standardized β coefficients are reported.

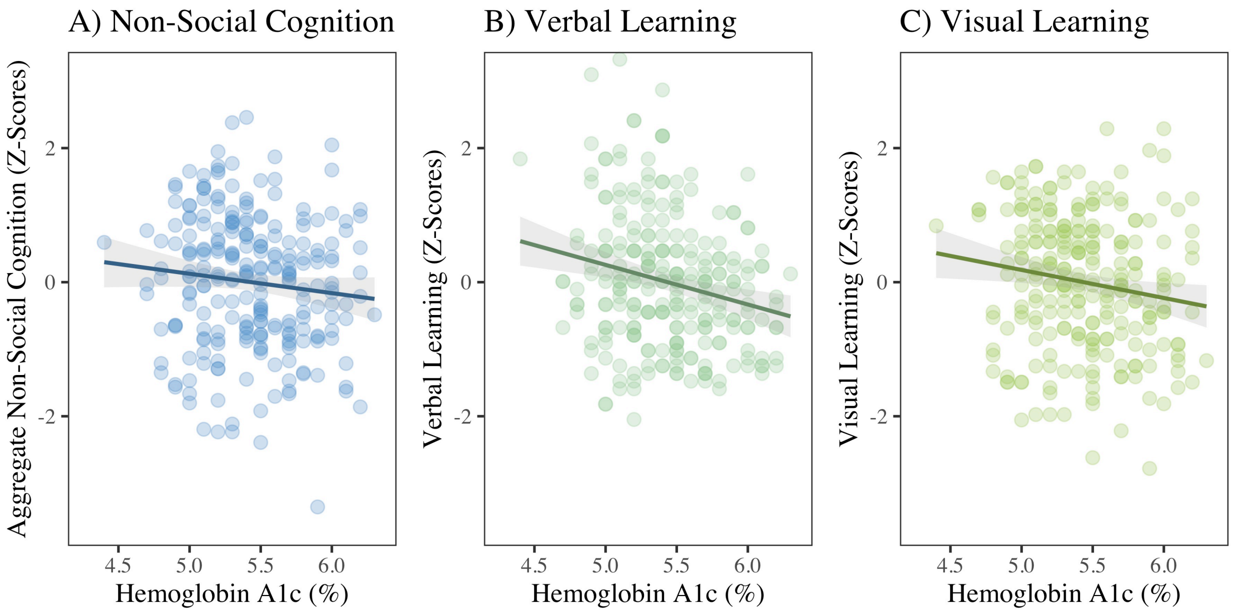


**Supplemental Figure 3 - Non-social Neurocognition and Hemoglobin A1c in SSD**: (A) Aggregate non-social neurocognition was not correlated with HbA1c ($\beta$=-0.11, p=0.10). (B) Verbal learning and memory was significantly related to HbA1c ($\beta$=-0.22, p<0.001). (C) Visual learning and memory was significantly related to HbA1c ($\beta$=-0.16, p=0.01). There was no significant relationship between HbA1c and processing speed, attention and vigilance, working memory, or reasoning. Standardized $\beta$ coefficients are reported.

**Supplemental Information on Waist Circumference**

The International Diabetes Federation (IDF) defines separate waist circumference cutoff points for individuals of Asian descent (Chinese, Japanese, and South Asian): 90cm for men and 80cm for women vs. individuals of European and other descent: 94cm for men and 80cm for women.[1]

Analysis using these criteria did not alter our main findings.

Overall social cognition continued to be more impaired among individuals with high waist circumference ($p=0.04$, Cohen's $d = -0.36$).

Cumulative metabolic risk, re-calculated using the IDF guidelines, continued to be significantly correlated with greater impairment in overall social cognition ($p<0.001$, $\beta = -0.37$). As before, the results remain significant when covarying for sex, race, and ethnicity ($p=0.005$, $\beta = -0.24$), but are no longer significant when covarying for nonsocial neurocognition.

1. Ford ES. Prevalence of the Metabolic Syndrome Defined by the International Diabetes Federation Among Adults in the U.S. *Diabetes Care*. 2005;28(11):2745-2749. doi:10.2337/diacare.28.11.2745
